# Significant contribution of the 3′→5′ exonuclease activity to the high fidelity of nucleotide incorporation catalyzed by human DNA polymerase ϵ

**DOI:** 10.1093/nar/gku1184

**Published:** 2014-11-20

**Authors:** Walter J. Zahurancik, Seth J. Klein, Zucai Suo

**Affiliations:** 1 Department of Chemistry and Biochemistry, The Ohio State University, Columbus, OH 43210, USA; 2 The Ohio State Biochemistry Program, The Ohio State University, Columbus, OH 43210, USA; 3 Department of Molecular Genetics, The Ohio State University, Columbus, OH 43210, USA

## Abstract

Most eukaryotic DNA replication is performed by A- and B-family DNA polymerases which possess a faithful polymerase activity that preferentially incorporates correct over incorrect nucleotides. Additionally, many replicative polymerases have an efficient 3′→5′ exonuclease activity that excises misincorporated nucleotides. Together, these activities contribute to overall low polymerase error frequency (one error per 10^6^–10^8^ incorporations) and support faithful eukaryotic genome replication. Eukaryotic DNA polymerase ϵ (Polϵ) is one of three main replicative DNA polymerases for nuclear genomic replication and is responsible for leading strand synthesis. Here, we employed pre-steady-state kinetic methods and determined the overall fidelity of human Polϵ (hPolϵ) by measuring the individual contributions of its polymerase and 3′→5′ exonuclease activities. The polymerase activity of hPolϵ has a high base substitution fidelity (10^−4^–10^−7^) resulting from large decreases in both nucleotide incorporation rate constants and ground-state binding affinities for incorrect relative to correct nucleotides. The 3′→5′ exonuclease activity of hPolϵ further enhances polymerization fidelity by an unprecedented 3.5 × 10^2^ to 1.2 × 10^4^-fold. The resulting overall fidelity of hPolϵ (10^−6^–10^−11^) justifies hPolϵ to be a primary enzyme to replicate human nuclear genome (0.1–1.0 error per round). Consistently, somatic mutations in hPolϵ, which decrease its exonuclease activity, are connected with mutator phenotypes and cancer formation.

## INTRODUCTION

DNA polymerases (Pols^1^) perform a wide variety of biological functions that are critical to the proliferation and maintenance of genomic DNA including DNA replication, DNA repair and translesion DNA synthesis. DNA polymerases are organized into seven families (A, B, C, D, X, Y and RT) and they share a structurally similar polymerase core consisting of finger, palm and thumb domains that together form a right-hand geometry (1–3). Besides the conserved polymerase core, DNA polymerases from different families possess additional domains and structural features that broaden their functional diversity *in vivo*. For instance, many replicative A- and B-family DNA polymerases possess a 3′→5′ exonuclease domain containing conserved carboxylate residues that are required for coordinating divalent metal ions to catalyze the excision of mismatched bases from the primer 3′ terminus (3–7).

Highly accurate DNA synthesis is critical for eukaryotic genome replication and stability. To ensure that DNA is faithfully copied from generation to generation, cells employ high-fidelity DNA polymerases that make only a single error per 10^6^–10^8^ nucleotide incorporation events (8–11). Kinetically, the polymerase active site alone in a replicative DNA polymerase has been found to exhibit a nucleotide selectivity of 10^4^–10^7^ (10–14). It was originally hypothesized that the amplification of free energy differences between correct and incorrect nucleotide incorporation by DNA polymerases was sufficient to account for the fidelity of DNA replication (15). More recently, the measured energetic difference between correct and incorrect nucleotide incorporation by three DNA polymerases account for most of the high fidelity displayed by these enzymes (16). Overall, nucleotide selection by DNA polymerases is guided by a wide variety of factors, such as base stacking (17), nucleotide desolvation (18), induced-fit conformational changes (14) and shape complementarity (17). In addition to the contributions of these factors to DNA polymerase fidelity, the 3′→5′ proofreading activity found in most A- and B-family DNA polymerases further improves the fidelity of DNA replication by as much as 200-fold (11,19,20).

In eukaryotes, three replicative DNA polymerases from the B-family, Polα, Polδ and Polϵ, are responsible for the majority of DNA replication (21). Human Polϵ (hPolϵ) is a heterotetramer, consisting of a catalytic subunit, p261, as well as three smaller subunits: p59, p12 and p17 (22). Though the structure of hPolϵ remains elusive, the crystal structure of the truncated catalytic subunit of yeast Polϵ (yPolϵ) was recently solved and shows the canonical right-hand configuration consisting of finger, thumb and palm domains in addition to an N-terminal domain and a 3′→5′ exonuclease domain. Surprisingly, the palm domain of yPolϵ was found to contain additional structural elements, including a previously unidentified ‘P domain’ which may play a role in aiding processive DNA synthesis catalyzed by Polϵ (23,24).

Genetic studies have shown that Polϵ is primarily responsible for synthesizing the leading strand during DNA replication (25–28). To serve this role effectively, Polϵ must be able to synthesize DNA efficiently and accurately. Recently, our lab utilized pre-steady-state kinetics to elucidate a minimal kinetic mechanism of correct nucleotide incorporation catalyzed by an exonuclease-deficient version of the N-terminal fragment (residues 1–1189) of the catalytic subunit p261 of hPolϵ (hPolϵ exo-) (29). Our studies reveal that hPolϵ inserts the correct nucleotide via an induced-fit mechanism and the rate-determining step is a protein conformational change step that occurs prior to phosphodiester bond formation. The proposed kinetic mechanism has been observed in most kinetically characterized DNA polymerases (8,30–36). For hPolϵ exo-, forward mutation assays estimated that it has a base substitution fidelity of 10^−5^, which is similar to the background of the assays and thus the error rate may even be overestimated (37). However, the overall fidelity of hPolϵ, as a function of its two enzymatic functions, has not yet been determined through pre-steady-state kinetic methods. In this paper, we determined the base substitution fidelity of hPolϵ exo- using pre-steady-state kinetic methods. Moreover, we investigated the contributions of mismatch extension and exonuclease activity to the overall fidelity of the wild-type, exonuclease-proficient N-terminal fragment of p261 of hPolϵ (hPolϵ exo+).

## MATERIALS AND METHODS

### Materials

The chemicals used for experiments were purchased from the following sources: [γ-^32^P]ATP from Perkin-Elmer Life Sciences (Boston, MA, USA); Optikinase from USB (Cleveland, OH, USA) and dNTPs from Bioline (Taunton, MA, USA). Both the wild-type (hPolϵ exo+) and the exonuclease-deficient triple mutant (D275A/E277A/D368A, hPolϵ exo-) forms of the truncated hPolϵ catalytic subunit were overexpressed and purified as described previously (29).

### DNA substrates

The DNA substrates listed in Table 1 were purchased from Integrated DNA Technologies, Inc. (Coralville, IA, USA) and purified as described previously (38). The 21- and 22-mer primer strands were 5′-radiolabeled by incubation with [γ-^32^P]ATP and Optikinase for 3 h at 37°C, and then purified from free [γ-^32^P]ATP by passing through a Bio-Spin 6 column (Bio-Rad). The 5′-radiolabeled primers were then annealed to the 41-mer templates by incubating the primer with a 1.15-fold excess of template at 95°C for 5 min before cooling slowly to room temperature over several hours.

**Table 1.** Sequences of DNA substrates

D-1	5′-CGCAGCCGTCCAACCAACTCA-3′
	3′-GCGTCGGCAGGTTGGTTGAGTAGCAGCTAGGTTACGGCAGG-5′
D-6	5′-CGCAGCCGTCCAACCAACTCA-3′
	3′-GCGTCGGCAGGTTGGTTGAGTGGCAGCTAGGTTACGGCAGG-5′
D-7	5′-CGCAGCCGTCCAACCAACTCA-3′
	3′-GCGTCGGCAGGTTGGTTGAGTTGCAGCTAGGTTACGGCAGG-5′
D-8	5′-CGCAGCCGTCCAACCAACTCA-3′
	3′-GCGTCGGCAGGTTGGTTGAGTCGCAGCTAGGTTACGGCAGG-5′
M-1	5′-CGCAGCCGTCCAACCAACTCAC-3′
	3′-GCGTCGGCAGGTTGGTTGAGTAGCAGCTAGGTTACGGCAGG-5′
M-7	5′-CGCAGCCGTCCAACCAACTCAC-3′
	3′-GCGTCGGCAGGTTGGTTGAGTTGCAGCTAGGTTACGGCAGG-5′
M-8	5′-CGCAGCCGTCCAACCAACTCAC-3′
	3′-GCGTCGGCAGGTTGGTTGAGTCGCAGCTAGGTTACGGCAGG-5′

### Polymerase and exonuclease single-turnover assays

All assays using hPolϵ exo- or hPolϵ exo+ were performed at 20°C in reaction buffer E (50 mM Tris-OAc, pH 7.4 at 20°C, 8 mM Mg(OAc)_2_, 1 mM DTT, 10% glycerol, 0.1 mg/ml bovine serum albumin and 0.1 mM ethylenediaminetetraacetic acid (EDTA)). Fast reactions were carried out using a rapid chemical quench-flow apparatus (KinTek). Notably, all reactions were performed at 20°C since the rate constant for correct nucleotide incorporation at 37°C was too fast (*k*_p_ > 500 s^−1^) to be measured accurately by using the rapid chemical quench-flow apparatus. For polymerization single-turnover assays, a pre-incubated solution of hPolϵ exo- (260 nM) and a 5′-radiolabeled DNA substrate (20 nM) in buffer E was rapidly mixed with Mg^2+^ (8 mM) and varying concentrations of dNTP. For exonuclease assays, a pre-incubated solution of hPolϵ exo+ (200 nM) and a 5′-radiolabeled DNA substrate (20 nM) in buffer E was rapidly mixed with Mg^2+^ (8 mM) in the absence of nucleotide to initiate the excision reaction. All reactions were quenched with the addition of 0.37 M EDTA. All reported concentrations are final. Most data, unless otherwise specified, were collected from single trials due to insufficient amount of hPolϵ to repeat each measurement in triplicate.

### Product analysis

Reaction products were separated by denaturing polyacrylamide gel electrophoresis (17% acrylamide, 8 M urea and 1× TBE running buffer) and quantified using a Typhoon TRIO (GE Healthcare) and ImageQuant (Molecular Dynamics).

### Data analysis

All kinetic data were fit by nonlinear regression using KaleidaGraph (Synergy Software). Data from polymerization assays under single-turnover conditions were fit to Equation (1)

(1) \begin{equation*} [{\rm product}] = A[1 - \exp ( - {\rm }k_{{\rm obs}} t)] \end{equation*}

where *A* is the amplitude of product formation and *k*_obs_ is the observed single-turnover rate constant.

Data from the plot of *k*_obs_ versus dNTP concentration were fit to Equation (2)

(2) \begin{equation*} k_{{\rm obs}} = k_{\rm p} [{\rm dNTP}]/(K_{\rm d} + [{\rm dNTP}]) \end{equation*}

where *k*_p_ is the maximum rate constant of nucleotide incorporation and *K*_d_ is the equilibrium dissociation constant for dNTP binding. When *K*_d_ is very large, the plot of *k*_obs_ versus dNTP concentration was fit to Equation (3)

(3) \begin{equation*} k_{{\rm obs}} = (k_{\rm p} /K_{\rm d} )[{\rm dNTP}] \end{equation*}

to yield the substrate specificity constant, *k*_p_/*K*_d._

Data from exonuclease assays under single-turnover conditions were fit to Equation (4)

(4) \begin{equation*} [{\rm product}] = A[{\rm exp}( - {\rm }k_{{\rm exo}} t)] + {\rm C} \end{equation*}

where *A* is the reaction amplitude and *k*_exo_ is the overall DNA excision rate constant.

All reported errors were generated by fitting the data to the above equations through Kaleidagraph.

## RESULTS

### Substrate specificity of hPolϵ exo-

**Figure 1.**
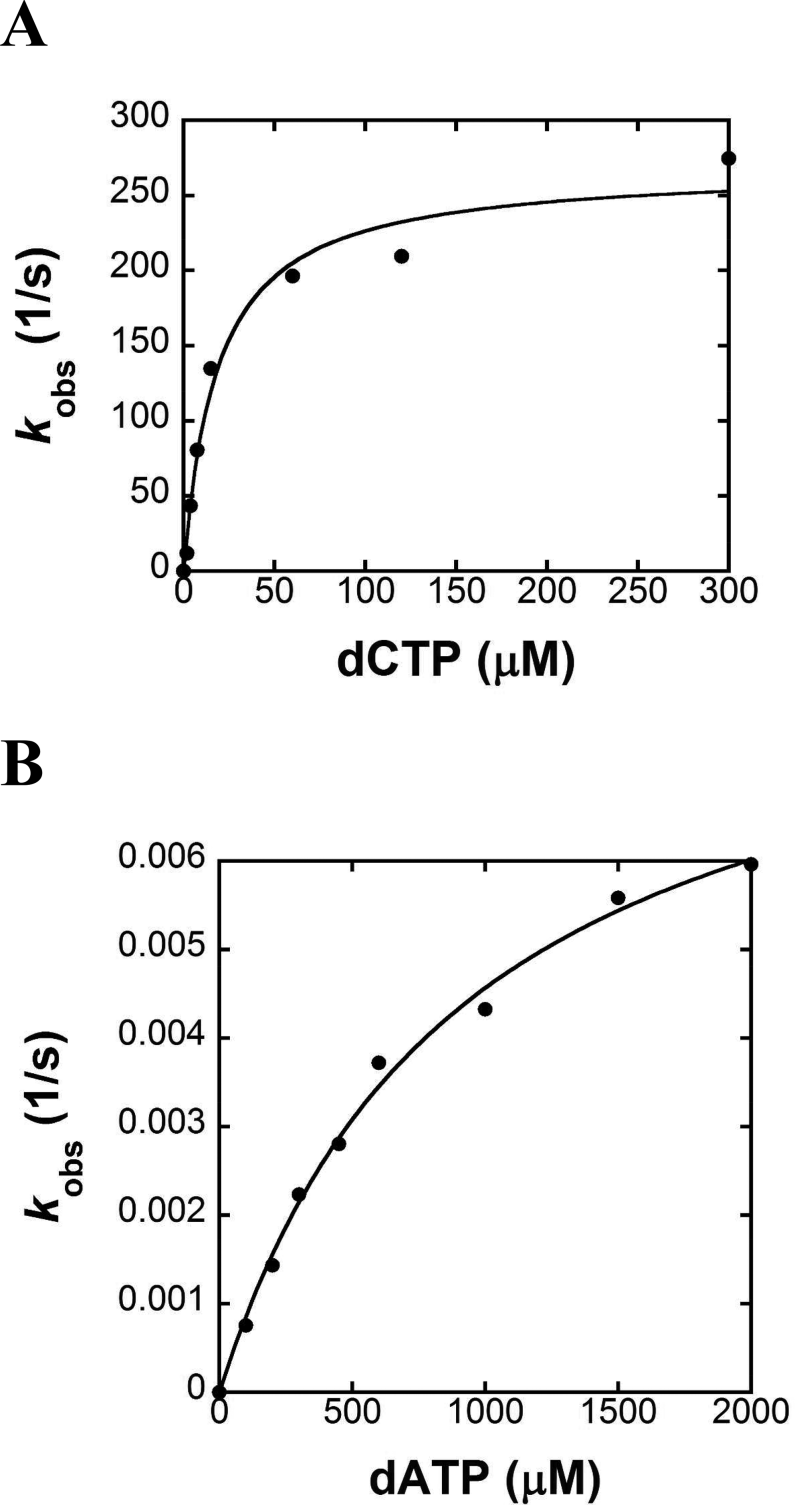
Nucleotide concentration dependence on the pre-steady-state kinetic parameters of correct dCTP and incorrect dATP incorporation opposite dG catalyzed by hPolϵ exo- at 20°C. (A) A pre-incubated solution of hPolϵ exo- (260 nM) and 5′-radiolabeled D-6 (20 nM) was mixed with increasing concentrations of correct dCTP and Mg^2+^ for various times. The plot of product concentration versus time was fit to Equation (1) to yield *k*_obs_ (data not shown). The resulting *k*_obs_ values were plotted against dCTP concentration and fit to Equation (2) to yield a *k*_p_ of 268 ± 14 s^−1^ and a *K*_d_ of 19 ± 4 μM; (B) hPolϵ exo- and 5′-radiolabeled D-6 were mixed with increasing concentrations of incorrect dATP and Mg^2+^ as described above. The data were similarly processed to yield a *k*_p_ of (8.8 ± 0.4) × 10^−3^ s^−1^ and a *K*_d_ of (9 ± 1) × 10^2^ μM.

In our recent publication we revealed through pre-steady-state kinetics that hPolϵ, like all other kinetically characterized polymerases, catalyzes correct nucleotide incorporation via an induced-fit mechanism (29). At 20°C, hPolϵ exo- binds and incorporates correct dTTP opposite dA with a maximum rate constant, *k*_p_, of 248 s^−1^ and an equilibrium dissociation constant, *K*_d_, of 31 μM (29). However, *k*_p_ and *K*_d_ for an incorrect incoming nucleotide have not yet been determined. We expected that hPolϵ, like other replicative DNA polymerases, exhibits high selectivity for correct incoming nucleotides versus incorrect nucleotides through the combination of both a faster incorporation rate constant and a higher ground-state binding affinity (1/*K*_d_). To confirm this hypothesis, we measured the substrate specificities (*k*_p_/*K*_d_) for each of the 15 remaining possible incoming nucleotide and templating base combinations through four perfectly matched DNA substrates (D-1, D-6, D-7 and D-8) listed in Table 1. As examples, the plots of *k*_obs_ versus dNTP concentration for the extension of the 21-mer primer in D-6 are shown for correct dCTP and incorrect dATP in Figure 1A and B, respectively. The plot in Figure 1A was fit to Equation (2) (see Materials and Methods) to obtain a *k*_p_ of 268 ± 14 s^−1^ and a *K*_d_ of 19 ± 4 μM as well as a calculated *k*_p_/*K*_d_ of 14 μM^−1^s^−1^ for correct dCTP incorporation. Likewise, the plot in Figure 1B was fit to Equation (2) to yield a *k*_p_ of (8.8 ± 0.4) × 10^−3^ s^−1^, a *K*_d_ of (9 ± 1) × 10^2^ μM and a *k*_p_/*K*_d_ of 9.8 × 10^−6^ μM^−1^s^−1^ for incorrect dATP incorporation. Similarly, the kinetic parameters for all other combinations of nucleotides and templating bases were determined at 20°C and are listed in Table 2. Notably, the *k*_p_ and *K*_d_ values for dCTP misincorporation opposite dC could not be determined due to the extremely weak binding affinity (>2 mM) of the incorrect dCTP. In this case, the plot of *k*_obs_ versus dCTP concentration (data not shown) was fit to a linear equation (Equation (3)) to give the corresponding *k*_p_/*K*_d_ value (1.5 × 10^−5^ μM^−1^ s^−1^, Table 2). Overall, the base substitution fidelity (*F*_pol_) of hPolϵ exo- was determined to be 10^−4^–10^−7^ (Table 2).

**Table 2.** Kinetic parameters for correct and incorrect nucleotide incorporation catalyzed by hPolϵ exo- at 20°C

dNTP	*k*_p_ (s^−1^)	*K*_d_ (μM)	*k*_p_/*K*_d_(μM^−1^s^−1^)	*F*_pol_^a^
Template dA (D-1)				
dTTP^b^	248 ± 6	31 ± 2	8	
dATP	0.61 ± 0.04	(6 ± 1) × 10^2^	1.0 × 10^−3^	1.2 × 10^−4^
dCTP	5.2 ± 0.9	(2.0 ± 0.6) × 10^3^	2.6 × 10^−3^	3.2 × 10^−4^
dGTP	(1.13 ± 0.04) × 10^−2^	(3.2 ± 0.3) × 10^2^	3.5 × 10^−5^	4.4 × 10^−6^
				
Template dG (D-6)				
dCTP	268 ± 14	19 ± 4	14	
dTTP	0.63 ± 0.06	(7 ± 2) × 10^2^	9.0 × 10^−4^	6.4 × 10^−5^
dATP	(8.8 ± 0.4) × 10^−3^	(9 ± 1) × 10^2^	9.8 × 10^−6^	7.0 × 10^−7^
dGTP	(8.6 ± 0.2) × 10^−2^	(2.4 ± 0.3) × 10^2^	3.6 × 10^−4^	2.6 × 10^−5^
				
Template dT (D-7)				
dATP	275 ± 12	33 ± 5	8	
dTTP	(4.7 ± 0.4) × 10^−2^	(9 ± 2) × 10^2^	5.2 × 10^−5^	6.5 × 10^−6^
dCTP	(7.4 ± 0.6) × 10^−2^	(1.1 ± 0.2) × 10^3^	6.7 × 10^−5^	8.4 × 10^−6^
dGTP	0.58 ± 0.06	(1.1 ± 0.2) × 10^3^	5.3 × 10^−4^	6.6 × 10^−5^
				
Template dC (D-8)				
dGTP	219 ± 13	9 ± 2	24	
dTTP	3.1 ± 0.3	(6 ± 1) × 10^2^	5.2 × 10^−3^	2.2 × 10^−4^
dATP	1.2 ± 0.1	(9 ± 2) × 10^2^	1.3 × 10^−3^	5.4 × 10^−5^
dCTP	-	-	1.5 × 10^−5^	6.2 × 10^−7^

^a^Calculated as (*k*_p_/*K*_d_)_incorrect_/[(*k*_p_/*K*_d_)_correct_ + (*k*_p_/*K*_d_)_incorrect_].

^b^Reference (29).

### Mismatch extension fidelity of hPolϵ exo-

**Figure 2.**
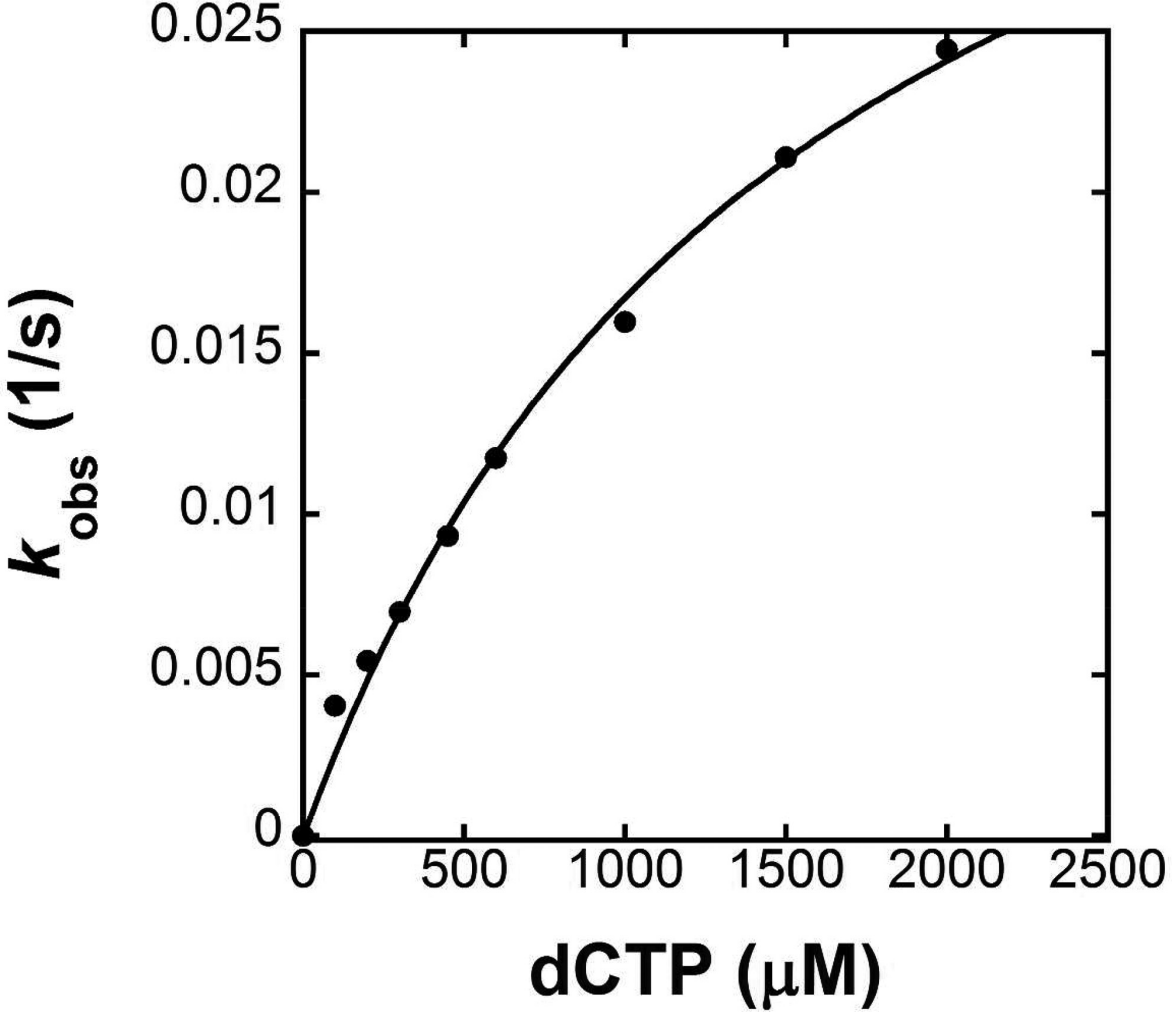
Extension of a mismatched base pair catalyzed by hPolϵ exo- at 20°C. A pre-incubated solution of hPolϵ exo- (260 nM) and 5′-radiolabeled M-7 (20 nM) was rapidly mixed with increasing concentrations of dCTP and Mg^2+^ for various times. The product concentration was plotted against time and fit to Equation (1) to yield *k*_obs_ (data not shown). The *k*_obs_ values were plotted against dCTP concentration and fit to Equation (3) to yield a *k*_p_ of (4.3 ± 0.4) × 10^−2^ s^−1^ and a *K*_d_ of (1.6 ± 0.2) × 10^3^ μM.

After a misincorporation event, hPolϵ will excise the nascent mismatched base pair, dissociate from the DNA substrate or further extend the mismatched base pair. Following selective inhibition of its 3′→5′ exonuclease activity by mutating three highly conserved carboxylate residues (D275/E277/D368) at the exonuclease active site to alanine (29), we were able to determine the *k*_p_/*K*_d_ values for the incorporation of both a correct nucleotide and an incorrect nucleotide on DNA substrates containing a single mismatched base at the primer 3′ terminus (M-1, M-7 and M-8 in Table 1). As an example, the plot of *k*_obs_ versus dCTP concentration for the extension of M-7 (Figure 2) was fit to Equation (2) (see Materials and Methods) to yield a *k*_p_ of (4.3 ± 0.4) × 10^−2^ s^−1^ and a *K*_d_ of (1.6 ± 0.2) × 10^3^ μM. Notably, M-7 contains a C:T mismatch at the primer–template junction, but is otherwise identical to the four correctly matched DNA substrates (D-1, D-6, D-7 and D-8 in Table 1). Interestingly, both correct dCTP and incorrect dGTP with M-7 had very low substrate specificities which were comparable to the values measured for incorrect nucleotide incorporation into a correctly matched DNA substrate (Table 3). Similarly, the kinetic parameters for correct dCTP and incorrect dGTP incorporation into the other two mismatched DNA substrates, M-1 and M-8, in Table 1 at 20°C were determined and are listed in Table 3.

**Table 3.** Kinetic parameters for mismatch extension and excision catalyzed by hPolϵ exo- and hPolϵ exo+ at 20°C

dNTP	*k*_p_ (s^−1^)	*K*_d_ (μM)	*k*_p_/*K*_d_(μM^−1^ s^−1^)	*F*_ext_^a^	*k*_obs_ (s^−1^)^b^	*k*_exo_ (s^−1^)	*F*_exo_^c^
C:A mismatch (M-1)							
dCTP	(4.0 ± 0.4) × 10^−2^	(5.4 ± 1.3) × 10^2^	7.4 × 10^−5^		6.2 × 10^−3^	-	
dGTP	(3.6 ± 0.3) × 10^−4^	(5.3 ± 1.3) × 10^2^	6.8 × 10^−7^	9.1 × 10^−3^	5.7 × 10^−5^	-	
-	-	-	-		-	2.2 ± 0.1	350
C:T mismatch (M-7)							
dCTP	(4.3 ± 0.4) × 10^−2^	(1.6 ± 0.2) × 10^3^	2.7 × 10^−5^		2.5 × 10^−3^	-	
dGTP	(6.3 ± 0.5) × 10^−4^	(6.4 ± 1.0) × 10^2^	9.8 × 10^−7^	3.5 × 10^−2^	8.5 × 10^−5^	-	
-	-	-	-		-	2.9 ± 0.3	1200
C:C mismatch (M-8)							
dCTP	-	-	2.6 × 10^−6^		2.6 × 10^−4^	-	
dGTP	(6.1 ± 0.3) × 10^−4^	(1.5 ± 0.1) × 10^3^	4.1 × 10^−7^	0.14	3.8 × 10^−5^	-	
-	-	-	-		-	3.0 ± 0.7	12 000

^a^Calculated as (*k*_p_/*K*_d_)_incorrect_/[(*k*_p_/*K*_d_)_correct_ + (*k*_p_/*K*_d_)_incorrect_].

^b^Calculated as *k*_p_[dNTP]/(*K*_d_ + [dNTP]) during extension from a mismatched primer terminus at an intracellular nucleotide concentration of 100 μM.

^c^Calculated as *k*_exo_/*k*_obs_.

### Excision of matched and mismatched DNA substrates by hPolϵ exo+

**Figure 3.**
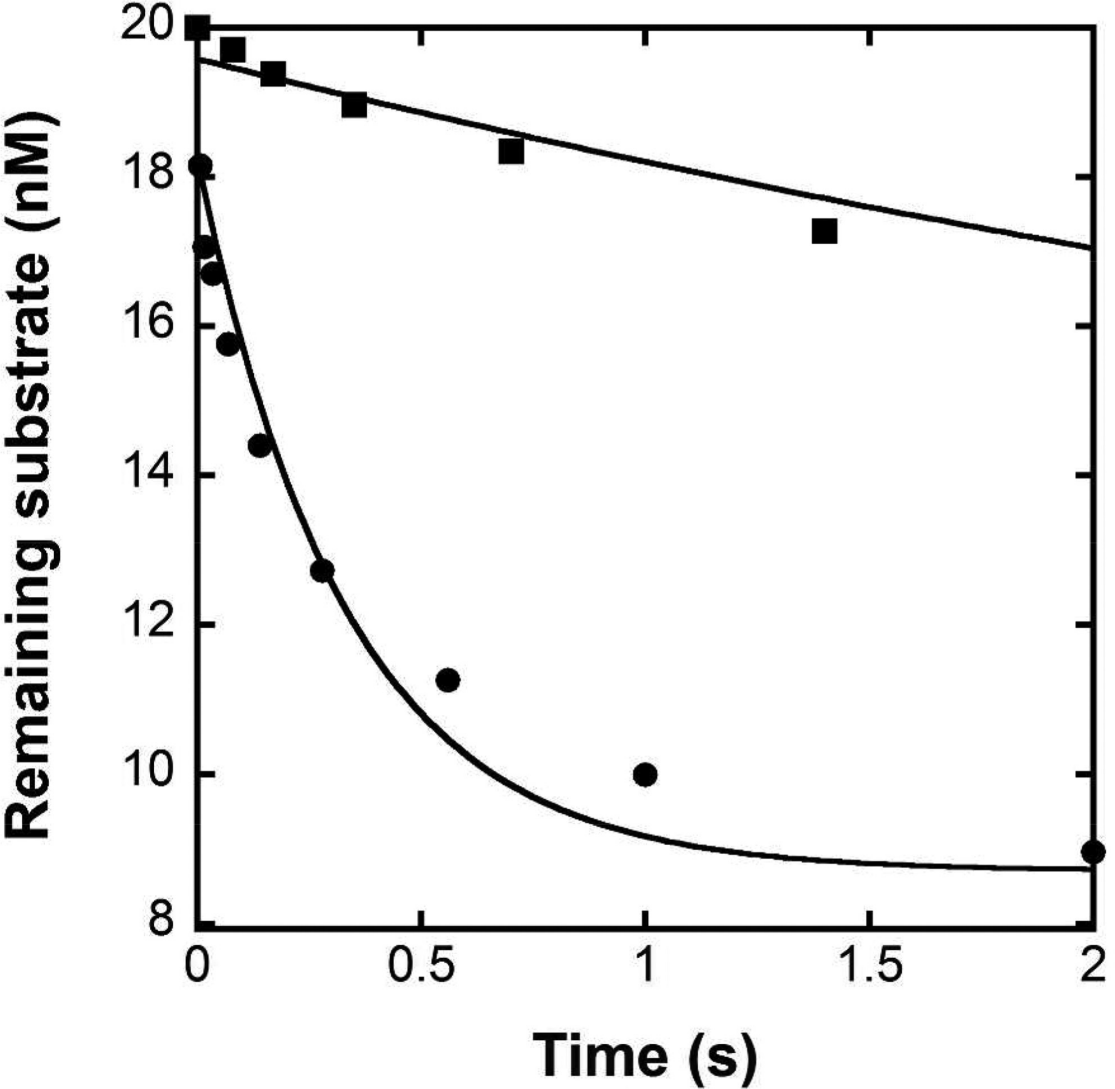
Excision of primers with matched and mismatched 3′ termini catalyzed by hPolϵ exo+ at 20°C. A pre-incubated solution of 200 nM of hPolϵ exo+ and 20 nM of 5′-radiolabeled D-8 (▪) or M-8 (•) was rapidly mixed with Mg^2+^ for various times before been quenched with 0.37 M EDTA. The remaining substrate concentration was plotted versus time and fit to Equation (4) to yield a *k*_exo_ of 0.17 ± 0.02 s^−1^ for the matched D-8 substrate and 3.0 ± 0.7 s^−1^ for the mismatched M-8 substrate.

hPolϵ, like most A- and B-family replicative DNA polymerases, possesses a 3′→5′ exonuclease proofreading activity that is proficient in removing mismatched bases from the primer 3′ terminus. It is expected that the exonuclease activity of hPolϵ will be kinetically favored over its polymerase activity in the presence of a mismatched primer terminus due to a significantly higher rate of excision versus extension. On the other hand, excision of a matched base pair should be much slower than correct nucleotide incorporation to prevent futile competition with 5′→3′ primer extension during processive DNA synthesis. To verify this hypothesis, we measured the overall excision rate constants (*k*_exo_) of matched versus mismatched base pairs by hPolϵ exo+. The D-8 and M-8 substrates (Table 1) were used to measure the *k*_exo_ values for a matched and mismatched primer–template pair, respectively. The concentration of remaining substrate was plotted versus time and the data were fit to Equation (4) (see Materials and Methods) to yield *k*_exo_ (Figure 3). The *k*_exo_ values were determined to be 0.17 ± 0.02 s^−1^ and 3.0 ± 0.7 s^−1^ for matched (D-8) and mismatched (M-8) primer–template pairs at 20°C, respectively. These measurements were repeated at a lower enzyme concentration and *k*_exo_ was found to be unaffected by the ratio of hPolϵ exo+ to DNA (data not shown). Notably, the measured *k*_exo_ is not the true excision rate constant at the exonuclease active site (*k*_x_) since it is a function of *k*_x_, the forward and backward transfer rates of the primer 3′-terminal nucleotides between the polymerase and exonuclease active sites, and DNA dissociation and rebinding rates from the exonuclease active site. Similarly, we measured *k*_exo_ for the mismatched DNA substrates M-1 and M-8 (Table 1) and the *k*_exo_ values are listed in Table 3. Interestingly, Table 3 shows that the overall rate constant of excision was not significantly affected by the identity of the 3′ mismatched base pair in a DNA substrate.

## DISCUSSION

To determine if hPolϵ synthesizes DNA with high fidelity as observed with other replicative DNA polymerases, we used pre-steady-state kinetics to measure the kinetic parameters of nucleotide incorporation and excision on both matched and single-base mismatched DNA substrates. First, we calculated the base substitution fidelity of hPolϵ exo- by measuring the *k*_p_ and *K*_d_ values at 20°C for all 16 possible combinations of incoming nucleotides and templating bases. Correct nucleotides were incorporated with an average *k*_p_ and *K*_d_ of 252 s^−1^ and 23 μM, respectively. The *k*_p_ values for incorrect nucleotide incorporation varied widely from (8.8 ± 0.4) × 10^−3^ s^−1^ to 5.2 ± 0.9 s^−1^ while the *K*_d_ values ranged between (2.4 ± 0.3) × 10^2^ to (2.0 ± 0.6) × 10^3^ μM. Strikingly, the *k*_p_ difference between correct and incorrect nucleotide incorporation [(*k*_p_)_correct_/(*k*_p_)_incorrect_] contrasts broadly, varying by one to four orders of magnitude. A similar result was previously obtained from pre-steady-state kinetic analysis of hPolγ exo- (13). Overall, hPolϵ exo- incorporated a correct nucleotide with a 48- to 3.0 × 10^4^-fold faster rate constant than an incorrect nucleotide, and bound a correct nucleotide with a 10- to 100-fold higher affinity. Thus, the differences in both *k*_p_ and *K*_d_ were major determinants of the base substitution fidelity of hPolϵ exo-, which was calculated to be 10^−4^–10^−7^ (Table 2). Similar kinetic patterns of incorrect nucleotide discrimination were determined for other highly accurate replicative DNA polymerases, including hPolγ, T7 DNA polymerase and RB69 DNA polymerase (13,14,39). Interestingly, all DNA polymerases including hPolϵ exo- (Table 2) possess sequence-dependent base substitution fidelity.

The fidelity of DNA synthesis catalyzed by replicative DNA polymerases is further enhanced by an associated 3′→5′ exonuclease proofreading activity that selectively excises mismatched base pairs. We calculated the contribution of proofreading (*F*_exo_) to the fidelity of DNA synthesis catalyzed by hPolϵ by taking the ratio of the overall rate constant of mismatch excision (*k*_exo_) versus the rate constant of mismatch extension at a typical intracellular nucleotide concentration of 100 μM (*k*_obs_). For example, in the case of correct dCTP incorporation onto the mismatched M-1 substrate by hPolϵ exo-, the *k*_p_ and *K*_d_ values were determined to be (4.0 ± 0.4) × 10^−2^ s^−1^ and of (5.4 ± 1.3) × 10^2^ μM, respectively (Table 3). Using Equation (2), *k*_obs_ was calculated to be 0.0062 s^−1^. For the same mismatched DNA substrate, the *k*_exo_ was measured to be 2.2 s^−1^ with hPolϵ exo+ (Table 3). Thus, the contribution of proofreading to the overall fidelity of hPolϵ was calculated to be ∼350-fold (Table 4). When factored together with the base substitution fidelity of hPolϵ exo- (10^−4^–10^−7^), the overall *in vitro* polymerization fidelity of hPolϵ was determined to be 10^−6^–10^−9^ with a C:A mismatch (M-1). It should be noted that incorrect incorporation over a mismatch is much slower and less efficient than correct incorporation and thus, misincorporations were not considered in the determination of *F*_exo_ (Table 3).

**Table 4.** Comparison of the contribution of 3′→5′ exonuclease activity to the overall fidelity of replicative DNA polymerases when encountering a single base mismatch in the staggering end of a DNA substrate

Polymerase	Mismatch	*k*_exo_ (s^−1^)	*k*_obs (s_^−1^)^a^	*F*_exo_^b^	Excision%^c^
hPolϵ^d^	C:A	2.2	6.2 × 10^−3^	350	99.719
	C:T	2.9	2.5 × 10^−3^	1200	99.914
	C:C	3.0	2.6 × 10^−4^	12 000	99.991
*S. solfataricus* PolB1^e^	A:A	1.86	0.012	160	99.359
hPolγ^f^	T:T	0.4	0.1	4	80.000
T7 DNA polymerase^g^	A:A	2.3	0.012	190	99.481

^a^Calculated as *k*_p_[dNTP]/(*K*_d_ + [dNTP]) during extension from a mismatched primer terminus at an intracellular nucleotide concentration of 100 μM.

^b^Calculated as *k*_exo_/*k*_obs_.

^c^Calculated as *k*_exo_/(*k*_exo_ + *k*_obs_) for a single base mismatch.

^d^This work (performed at 20°C).

^e^Reference (11) (performed at 37°C).

^f^Reference (20) (performed at 37°C).

^g^Reference (19) (performed at 20°C).

Interestingly, the substrate specificity for the next correct nucleotide with hPolϵ exo- varied widely depending on the identity of the single base mismatch (Table 3). A similar result was obtained for *Escherichia coli* Klenow fragment which catalyzed mismatch extension with a rate constant that differed by as many as three orders of magnitude in a sequence-dependent manner (40). In contrast, the overall rate constant of mismatch excision by hPolϵ exo+ is not significantly affected. This is comparable to the observation that the rate constant of excision of a single base mismatch catalyzed by hPolγ is independent of mismatch identity (20). As a consequence of both a highly variable extension rate constant and a similar excision rate constant, the 3′→5′ exonuclease activity of hPolϵ appears to enhance its overall fidelity by two to four orders of magnitude based on the mismatched bases (Table 3). For better comparison, the *F*_exo_ values were calculated for several other replicative DNA polymerases (Table 4). Notably, the *F*_exo_ values are much larger with hPolϵ exo+ than with *Sulfolobus solfataricus* PolB1, hPolγ and T7 DNA polymerase and this is beneficiary to faithful replication of the vast nuclear human genome. However, the rate constants listed for extension of a primer containing a single base mismatch by *S. solfataricus* PolB1, hPolγ and T7 DNA polymerase in Table 4 were determined only for one specific mismatched base pair. Therefore, it is possible that the *F*_exo_ for these replicative DNA polymerases, as observed with hPolϵ exo+, varies in a large range depending on the identity of the single base mismatch.

Though the 3′→5′ proofreading activity of hPolϵ is highly efficient at removing mismatched base pairs, the possibility that hPolϵ may partition toward removal of a correctly matched base pair must be considered. For example, the extension rate constant (*k*_p_) on the D-8 substrate in the presence of the next correct nucleotide, dGTP, was measured to be 219 ± 13 s^−1^ (Table 2), while the overall excision rate constant (*k*_exo_) was 0.17 ± 0.02 s^−1^ (Figure 3). Since typical cellular nucleotide concentrations (100 μM) are significantly higher than the *K*_d_ value (9 μM, Table 2) for dGTP with D-8, the dGTP incorporation rate constant should approach *k*_p_. Thus, the probability of matched base pair excision, given by *k*_exo_/(*k*_exo_ + *k*_p_), was calculated to be only 0.08% while the probability of further extension *k*_p_/(*k*_p_ + *k*_exo_) approached 100%. In contrast, for a single base mismatched terminus in a DNA substrate, the kinetic partitioning between excision *k*_exo_/(*k*_exo_ + *k*_obs_) and extension *k*_obs_/(*k*_obs_ + *k*_exo_) was calculated to be 99.719–99.991% and 0.009–0.281%, respectively (Table 4). Thus, the 3′→5′ proofreading activity of hPolϵ is very efficient at removing mismatched nucleotides without interfering with continuous faithful DNA synthesis.

From the combined contributions of both high polymerase selectivity (10^−4^–10^−7^, Table 2) and efficient 3′→5′ proofreading activity (3.5 × 10^2^ to 1.2 × 10^4^, Table 3), hPolϵ exhibits overall polymerization fidelity of 10^−6^–10^−11^ *in vitro*. Such high fidelity of DNA synthesis qualifies hPolϵ as a main enzyme to catalyze accurate replication of large human nuclear genome (3 × 10^9^ base pairs). As the key polymerase responsible for leading strand synthesis during nuclear genomic replication, hPolϵ must synthesize long stretches of DNA without making an error. Consistently, the fidelity of DNA replication in normal human cells was estimated to be 10^−9^–10^−10^ (41–43). Strikingly, somatic mutations in the 3′→5′ exonuclease domain of hPolϵ impair the proofreading activity, cause a high frequency of errors (>10^−4^ mutations per base) in the leading strand, elevate recurrent nonsense mutation rates in key tumor suppressors, such as TP53, ATM and PIK3R1, and ultimately lead to the formation of various cancers (27). This error frequency is greater than the high end of the fidelity range of hPolϵ exo- (10^−4^–10^−7^) measured here. Such a discrepancy suggests other cellular factors also contribute to the high leading strand mutation rate in tumors carrying inactivating mutations of the proofreading domain of hPolϵ.

Notably, the lower limit (10^−6^) of the fidelity range of hPolϵ (10^−6^–10^−11^) is significantly higher than the error frequency of normal human genome replication (10^−9^–10^−10^) (41–43). It is likely that this difference is accounted for by post-replication mismatch repair *in vivo*, which enhances replication fidelity by one to three orders of magnitude in *E. coli* and *Saccharomyces cerevisiae* (43–47). Additionally, it is possible that interactions between the p261 catalytic subunit and the smaller subunits or other proteins in the replisome may further enhance the fidelity of DNA replication *in vivo*. To investigate this hypothesis, we are currently studying the effect of the smaller subunits on the catalytic properties of p261 of hPolϵ.
